# Safety and effectiveness of acupuncture for POSEIDON patients in IVF/ICSI: A protocol for systematic review and meta-analysis

**DOI:** 10.1097/MD.0000000000022768

**Published:** 2020-10-16

**Authors:** Xinyun Zhu, Lijie Yang, Zimeng Li, Zhengqi Pan, Shijie Huang, Yueheng Xiong, Jie Wu

**Affiliations:** a Acupuncture and Moxibustion School Chengdu University of Traditional Chinese Medicine; b Hospital of Chengdu University of Traditional Chinese Medicine, Sichuan, China.

**Keywords:** acupuncture, ICSI, IVF, POSEIDON, protocol, systematic review

## Abstract

**Introduction:**

The purpose of this paper is to evaluate the efficacy and safety of acupuncture for POSEIDON patients undergoing IVF/ICSI.

**Methods:**

and analysis We will electronically search Pubmed, Medline, Embase, Web of Science, the Cochrane Central Register of Controlled Trial, China National Knowledge Infrastructure, China Biomedical Literature Database, China Science Journal Database and Wan-fang Database from their inception. Also, we will manually retrieve other resources, including reference lists of identified publications, conference articles, and grey literature. The clinical randomized controlled trials or quasi randomized controlled trials related to acupuncture treatment for POSEIDON patients in IVF/ICSI will be included in the study. The language is limited to Chinese and English. Research selection, data extraction, and research quality assessment will be independently completed by two researchers. Data were synthesized by using a fixed effect model or random effect model depend on the heterogeneity test. The clinical pregnancy rate (CPR) and live birth rate (LBR) will be the primary outcomes. The ongoing pregnancy, miscarriage rate (MR) and adverse events will also be assessed as secondary outcomes. RevMan V.5.3 statistical software will be used for meta-analysis, and the level of evidence will be assessed by Grading of Recommendations Assessment, Development, and Evaluation (GRADE). Continuous data will be expressed in the form of weighted mean difference or standardized mean difference with 95% confidence intervals (CIs), while dichotomous data will be expressed in the form of relative risk with 95% CIs.

**Ethics and dissemination:**

The protocol of this systematic review (SR) does not require ethical approval because it does not involve humans. We will publish this article in peer-reviewed journals and presented at relevant conferences.

**Systematic review registration:**

OSF Registries, DOI: 10.17605/OSF.IO/6WP2F (https://osf.io/6wp2f)

## 1 Introduction

In the treatment of infertility, in vitro fertilization (IVF) and intracytoplasmic sperm injection (ICSI) are major methods of assisted reproductive technology (ART).^[1]^ Retrieving adequate oocytes is essential for a satisfactory result in IVF/ICSI. However, patients with a diminished ovarian reserve (DOR) or poor ovarian response (POR) reports lower success rate, and the management of these patients is still in dilemma.^[2,3]^ To provide enough clinical recommendations for managing POR patients, the Patient-Oriented Strategies Encompassing IndividualizeD Oocyte Number (POSEIDON) criteria was proposed in 2016.^[4,5]^

The POSEIDON criteria classify POR patients into 4 groups according to former IVF cycle results, age, antral follicle count (AFC), and anti-mullerian hormone (AMH): Group 1: Patients <35 years with sufficient prestimulation ovarian reserve parameters (AFC ≥5, AMH ≥1.2 ng/mL) and with an unexpected poor or suboptimal ovarian response; Group 2: Patients ≥35 years with sufficient prestimulation ovarian reserve parameters (AFC ≥5, AMH ≥1.2 ng/mL) and with an unexpected poor or suboptimal ovarian response; Group 3: Patients <35 years with poor ovarian reserve prestimulation parameters (AFC <5, AMH <1.2 ng/mL); Group 4: Patients ≥35 years with poor ovarian reserve prestimulation parameters (AFC <5, AMH <1.2 ng/mL).^[4,5]^ With this classification, studies analyzed the results of completed IVF/ICSI cycles of POR patients, showing that in cumulative live birth rates (CLBR), optimistic outcomes were observed in group 1, 2, and 3.^[6,7]^ Thus, POSEIDON criteria might provide a more detailed and precise model in predicting IVF/ICSI prognosis on POR women.^[8]^

As a major form of complementary and alternative medicine, acupuncture is widely used before and during IVF/ICSI procedures^[9]^; however, its benefit is still uncertain.^[10–18]^ Meanwhile, on POR patients undergoing IVF/ICSI, acupuncture is reported effective in improving AMH, retrieved oocytes number, and clinical pregnancy rates (CPR).^[19]^ Under these circumstances, it is worthy to investigate acupuncture's efficacy on POR women with POSEIDON classification; thus, we would like to conduct this systematic review (SR) to evaluate acupuncture's influence on CPR, live birth rates (LBRs) for POSEIDON patients underwent IVF/ICSI.

## 2 Methods

The protocol has been registered on OSF as Registration DOI: 10.17605/OSF.IO/YU9T5 (https://osf.io/yu9t5). The protocol follows the Preferred Reporting Items for Systematic Reviews and Meta-Analyses Protocols (PRISMA-P) 2015 statement guidelines.^[20]^ We will report the changes in the full review if necessary.

### 2.1 Inclusion and exclusion criteria for study selection

#### 2.1.1 Inclusion criteria

This study will include randomized controlled trials (RCTs) of acupuncture as additional treatment for POSEIDON patients in IVF/ICSI, whether using blind method or allocation concealment method. The language of the trials to be included should be Chinese or English.

#### 2.1.2 Exclusion criteria

Following studies would be excluded: patient age <18 years, preimplantation genetic diagnosis/screening (PGD/PGS) included, case reports and reviews, literature not in English or Chinese language, clinical research studies that compared different kinds of acupuncture or moxibustion, besides standard IVF/ICSI, the treatment was combined with other treatments than acupuncture, and animal studies.

### 2.2 Types of participants

Patients meeting POSEIDON criteria, undergoing IVF/ICSI, all participants should accept acupuncture treatment. Patients under 18 years’ old would be excluded.

### 2.3 Types of interventions

The acupuncture treatment should be an additional treatment before or during IVF/ICSI, including traditional acupuncture, electro-acupuncture, laser acupuncture, auricular acupuncture, and so on; the details of acupuncture treatment should be clearly illustrated according to STRICTRA,^[21]^ involving the needle selection, acupoints selection, manipulations, course, among others.

### 2.4 Control

The control interventions include routine IVF/ICSI alone or routine IVF/ICSI plus sham/placebo acupuncture. Meanwhile, the control group should receive same IVF/ICSI treatment as acupuncture group.

The following studies would be excluded: RCTs which compare different kinds of acupuncture, placebo control or waiting list control, and acupuncture group and control group received different IVF/ICSI treatment.

### 2.5 Types of outcome measures

#### 2.5.1 Primary outcomes

We select CPR and LBR as our primary outcomes.

#### 2.5.2 Secondary outcomes

We also care about the following indexes: ongoing pregnancy, miscarriage rate (MR: [CPR-LBR]/CPR), and reported adverse events.

## 3 Data sources

### 3.1 Electronic searches

Following databases will be searched: PubMed, Web of Science, the Cochrane Central Register of Controlled Trials, AMED, MEDLINE, EMBASE, Cochrane Library, China National Knowledge Infrastructure (CNKI), Wanfang data, Chinese Scientific Journals Database (VIP), and China biomedical literature database (CBM). We will select the eligible studies published up to August 31, 2020. The search terms used in the SR are as follows: acupuncture, POSEIDON, poor ovarian response, IVF, and ICSI. We will not apply any language, population, or national restrictions. The specific search strategy will be (taking PubMed as an example) listed on Table 1. Similar search strategy will be applied to other electronic databases.

**Table 1:**
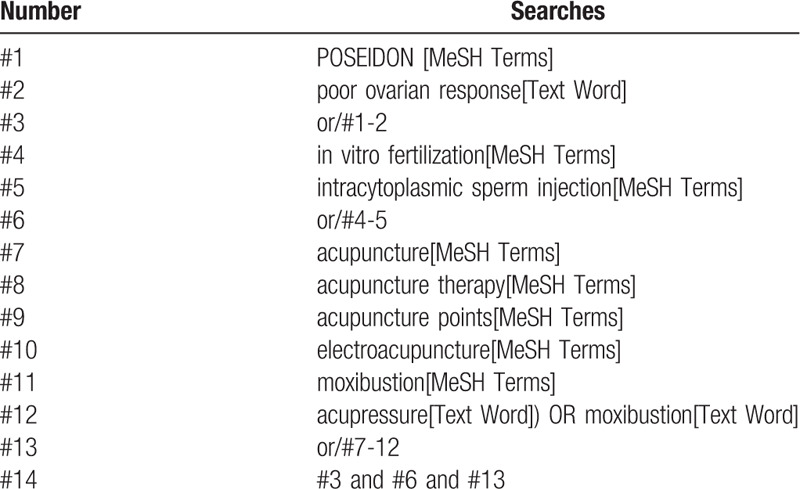
Search strategy sample of PubMed.

We will identify relevant randomized controlled trials and the selected studies will be analyzed according to the Cochrane Handbook.

### 3.2 Searching other resources

**Figure 1:**
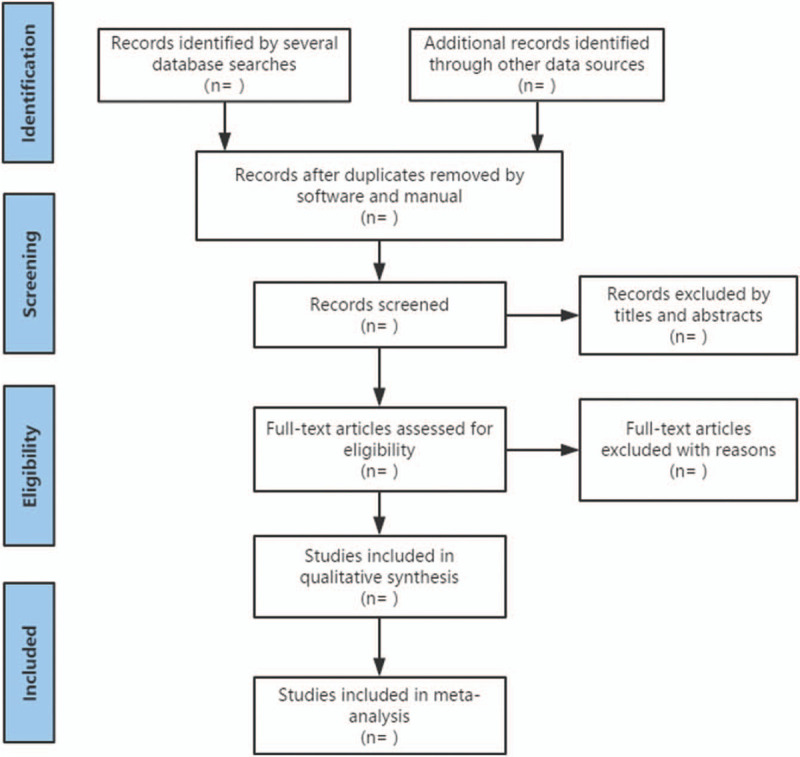
The research flow chart.

We also retrieve manual related documents, such as replacing and supplementing some reference documents, medical textbooks, clinical laboratory manuals, and the World Health Organization International Registry of Clinical Trials (ICTRP). At the same time, we will contact experts and authors in this field to obtain important information that cannot be found in the search. The research flow chart is shown in Figure 1.

## 4 Data collection and analysis

### 4.1 Selection of studies

Two independent researchers (ZX and YL) will assess the full-text articles from the search results independently against the inclusion and exclusion criteria. Discrepancies will be discussed and resolved by consensus with a third author (LZ).

### 4.2 Data extraction and management

The following information will be extracted from each study: research number, data extractor, date of data extraction, general situation of the study, research methodology, research population, baseline comparability, interventions, main outcome indicators, secondary outcome indicators, combined drug use, adverse reactions or complications, and so on. For those with questions or incomplete information, we will try to contact the author to obtain information before deciding whether to include it.

### 4.3 Assessment of the reporting quality and risk of bias

Two of the authors (ZX and YL) individually assessed the risk of bias using assessments included in the study were evaluated in the Cochrane System Evaluator's Manual for RCT quality evaluation criteria. Assessing the risk of bias: random sequence generation; allocation concealment; blinding of participants and personnel; blinding of outcome assessment; incomplete outcome data; selective outcome reporting; other bias. Every domain was classified as high risk of bias, low risk of bias, or unclear risk of bias. Any arising difference was resolved by discussion.

### 4.4 Measures of a treatment effect

We will measure continuous data with mean difference (MD) or standard MD (SMD) for the therapeutic effect with 95% CIs. For dichotomous data, risk ratios (RR) with 95% CIs will be calculated.

### 4.5 Management of missing data

To obtain the missing data, we will contact the corresponding author. If no response will be obtained, we will analyze only the available data and describe the reason and impact of this exclusion in the article.

### 4.6 Assessment of a reporting bias

Publication bias will be explored through funnel plot analysis. GRADE profiler 3.6 is used to evaluate the quality of evidence. The specific contents include: limitations of research, inconsistency of research results, indirect evidence, inadequate accuracy, publication bias. Finally, the quality of evidence is divided into 4 levels: high-level evidence, intermediate evidence, low-level evidence, and very low-level evidence.

### 4.7 Assessment of heterogeneity

All literature will use *I*^2^ value of the *χ*^2^ test (a = 0.1) to determine the heterogeneity. When *I*^2^ ≤50%, it is considered acceptable. When *I*^2^ > 50%, subgroup analysis should be performed to identify potential causes and record them.

### 4.8 Data synthesis and grading of quality of evidence

Reedman 5.3 software was used for statistical analysis of data. RR was used for binary variables and MD was used for continuous variables. Heterogeneity analysis will be conducted by heterogeneity test; *P* and *I*^2^ represent the size of heterogeneity among multiple studies. When *P* is >0.1 and *I*^2^ is <50%, it suggests heterogeneity is small; on the contrary, it suggests heterogeneity is large. Heterogeneity is mainly handled by subgroup analysis. Sensitivity analysis is used to test the reliability of the overall effect.

### 4.9 Subgroup analysis

When the heterogeneity test results are heterogeneous, we will conduct subgroup analysis to explore the possible causes of heterogeneity. The effects of different types of acupuncture therapy including design scheme, severity of illness, age, sex, and mild or severe AP were analyzed. We will also delete low-quality and/or medium-quality studies to check the robustness of the results.

### 4.10 Sensitivity analysis

Sensitivity analysis will be used to test the quality of the research contained in the sampled documents. The stability of the conclusions can be tested by re-analyzing the conclusions by inputting missing data and changing the type of research.

### 4.11 Ethics and dissemination

The results of the system review will be published in peer-reviewed journals, disseminated at relevant meetings, or disseminated in peer-reviewed publications, and we use aggregated published data to exclude individual patient data, so ethical approval, and informed consent are not required.

## 5 Discussion

Studies on acupuncture's influence in ART started in 2002,^[22]^ different RCTs and SRs reported conflicting results on acupuncture for CPR and LBR in IVF.^[9,11,16,18,23–27]^ Evidences showed, several acupuncture sessions performed on or around the day of embryo transfer are insufficient interventions to improve IVF birth outcomes, more sessions are associated with increases in clinical pregnancy and LBRs.^[28]^ Meanwhile, standardized, large sample trials based on TCM theories are still in lack to evaluate acupuncture in IVF.^[27]^

As a matter of fact, when informed IVF is in need, infertile women are stressful and hoping to find a therapy to improve IVF outcomes for themselves and for other infertile women,^[29]^ under this circumstance, acupuncture is widely used by individuals undergoing IVF^[30,31]^; it is reported to ameliorate stressful emotions during IVF procedures,^[13,32,33]^ reduce pain intensity and analgesic consumption,^[15]^ modulate neuroendocrine factors and immune factors, increase blood flow to the uterus and ovaries,^[34]^ promote the absorption of endometrial cavity fluid, and improve the uterine environment and endometrial receptivity.^[35]^ Meanwhile, when applied around the time of embryo transfer, acupuncture may be effective when compared with no adjunctive treatment with increased clinical pregnancies.^[36]^ Furthermore, for women experienced repeated implantation failure, acupuncture significantly improved the clinical outcomes of subsequent IVF cycles.^[37]^

In some women with DOR, POR might occur, it manifests as a reduction in follicular response, resulting in a reduced number of retrieved oocytes.^[2]^ These patients received worse clinical results from IVF cycles, and there lacked effective prediction and management.^[3,38]^ For better clinical decisions of these patents, POSEIDON criteria were carried out in 2016,^[4,5]^ dividing low prognosis patients into 4 groups with their age, AMH, AFC, and retrieved oocytes number of previous cycle. Retrospective studies suggested significant differences on CPR and LBR among 4 groups^[6,7]^; these results might be adequate references for building a predicting model in managing POR patients.

For POR patients in IVF, acupuncture was reported to improving endocrine hormone levels,^[17]^ including notably decreased levels of FSH, LH, and E2, and significantly increased AMH and AFC. Another study suggested acupuncture during in vitro fertilization and embryo transplantation could improve the clinical pregnancy rate on women with decreased ovarian reserve.^[39]^

Meanwhile, SRs^[19,27,40]^ concluded that acupuncture is beneficial for women with POR or with previous unsuccessful IVF attempts. With POSEIDON criteria further classifying POR patients, to investigate acupuncture's influence in these 4 groups respectively, we plan to conduct this SR, then provide references for the use of acupuncture on POSEIDON patients.

## Author contributions

**Conceptualization:** Xinyun Zhu, Zimeng Li.

**Data curation:** Xinyun Zhu, Zhengqi Pan.

**Formal analysis:** Xinyun Zhu, Lijie Yang.

**Funding acquisition:** Jie Wu.

**Methodology:** Zimeng Li.

**Project administration:** Yueheng Xiong, Jie Wu.

**Software:** Zimeng Li, Shijie Huang.

**Supervision:** Jie Wu.

**Validation:** Lijie Yang, Zimeng Li.

**Visualization:** Shijie Huang, Yueheng Xiong.

**Writing – original draft:** Xinyun Zhu, Lijie Yang.

**Writing – review & editing:** Zhengqi Pan, Shijie Huang.
